# Disentangling the taxonomy of the subfamily Rasborinae (Cypriniformes, Danionidae) in Sundaland using DNA barcodes

**DOI:** 10.1038/s41598-020-59544-9

**Published:** 2020-02-18

**Authors:** Arni Sholihah, Erwan Delrieu-Trottin, Tedjo Sukmono, Hadi Dahruddin, Renny Risdawati, Roza Elvyra, Arif Wibowo, Kustiati Kustiati, Frédéric Busson, Sopian Sauri, Ujang Nurhaman, Edmond Dounias, Muhamad Syamsul Arifin Zein, Yuli Fitriana, Ilham Vemendra Utama, Zainal Abidin Muchlisin, Jean-François Agnèse, Robert Hanner, Daisy Wowor, Dirk Steinke, Philippe Keith, Lukas Rüber, Nicolas Hubert

**Affiliations:** 1 Instut Teknologi Bandung, School of Life Sciences and Technology, Bandung, Indonesia; 2 UMR 5554 ISEM (IRD, UM, CNRS, EPHE), Université de Montpellier, Place Eugène Bataillon, 34095, Montpellier, cedex 05 France; 3 Museum für Naturkunde, Leibniz-Institut für Evolutions und Biodiversitätsforschung an der Humboldt-Universität zu Berlin, Invalidenstrasse 43, Berlin, 10115 Germany; 4 Universitas Jambi, Department of Biology, Jalan Lintas Jambi - Muara Bulian Km15, 36122 Jambi, Sumatra Indonesia; 5 Division of Zoology, Research Center for Biology, Indonesian Institute of Sciences (LIPI), Jalan Raya Jakarta Bogor Km 46, Cibinong, 16911 Indonesia; 6 Department of Biology Education, STKIP PGRI Sumatera Barat, Jl Gunung Pangilun, Padang, 25137 Indonesia; 7 Universitas Riau, Department of Biology, Simpang Baru, Tampan, Pekanbaru 28293 Indonesia; 8 Southeast Asian Fisheries Development Center, Inland Fisheries Resources Development and Management Department, 8 Ulu, Seberang Ulu I, Palembang 30267 Indonesia; 9 Research Institute for Inland Fisheries and Fisheries extensions, Agency for Marine and Fisheries Research, Ministry of Marine Affairs and Fisheries., Jl. H.A. Bastari No. 08, Jakabaring, Palembang 30267 Indonesia; 10 Universitas Tanjungpura, Department of Biology, Jalan Prof. Dr. H. Hadari Nawawi, Pontianak, 78124 Indonesia; 11 UMR 7208 BOREA (MNHN-CNRS-UPMC-IRD-UCBN), Muséum National d’Histoire Naturelle, 43 rue Cuvier, 75231, Paris, cedex 05 France; 12 UMR 5175 CEFE (IRD, UM, CNRS, EPHE), 1919 route de Mende, 34293, Montpellier, cedex 05 France; 13 Syiah Kuala University, Faculty of Marine and Fisheries, Banda, Aceh 23111 Indonesia; 14 Department of Integrative Biology, Centre for Biodiversity Genomics, 50 Stone Rd E, Guelph, ON N1G2W1 Canada; 15 Naturhistorisches Museum Bern, Bernastrasse 15, Bern, 3005 Switzerland; 16 Aquatic Ecology and Evolution, Institute of Ecology and Evolution, University of Bern, 3012 Bern, Switzerland

**Keywords:** Ichthyology, Phylogenetics

## Abstract

Sundaland constitutes one of the largest and most threatened biodiversity hotspots; however, our understanding of its biodiversity is afflicted by knowledge gaps in taxonomy and distribution patterns. The subfamily Rasborinae is the most diversified group of freshwater fishes in Sundaland. Uncertainties in their taxonomy and systematics have constrained its use as a model in evolutionary studies. Here, we established a DNA barcode reference library of the Rasborinae in Sundaland to examine species boundaries and range distributions through DNA-based species delimitation methods. A checklist of the Rasborinae of Sundaland was compiled based on online catalogs and used to estimate the taxonomic coverage of the present study. We generated a total of 991 DNA barcodes from 189 sampling sites in Sundaland. Together with 106 previously published sequences, we subsequently assembled a reference library of 1097 sequences that covers 65 taxa, including 61 of the 79 known Rasborinae species of Sundaland. Our library indicates that Rasborinae species are defined by distinct molecular lineages that are captured by species delimitation methods. A large overlap between intraspecific and interspecific genetic distance is observed that can be explained by the large amounts of cryptic diversity as evidenced by the 166 Operational Taxonomic Units detected. Implications for the evolutionary dynamics of species diversification are discussed.

## Introduction

Over the past two decades, the spectacular aggregation of species in biodiversity hotspots has attracted attention by scientists and stakeholders alike^1–4^. However, this exceptional concentration of often-endemic species at small spatial scales is threatened by the rise of anthropogenic disturbances. Of the 26 initially identified terrestrial biodiversity hotspots^1^, the ones located in Southeast Asia (SEA) (Indo-Burma, Philippines, Sundaland and Wallacea) currently rank among the most important both in terms of species richness and the extend of endemism but also rank as the most threatened by human activities^3^. Sundaland is currently the most diverse terrestrial biodiversity hotspot of SEA and is the most threatened^5^. Sundaland comprises Peninsular Malaysia and the islands of Java, Sumatra, Borneo, and Bali and its diversity originates from the complex geological history of the region, linked to major tectonic changes in the distribution of land and sea during the last 50 Million years (My)^6^, but also from eustatic fluctuations that have sporadically connected and disconnected Sundaland landmasses during glacial-interglacial cycles in the Pleistocene^7–9^. Therefore, Sundaland biogeography has received increased attention over the past decade resulting in the detection of contrasting spatial and temporal patterns in various groups^9–14^.

**Figure 1:**
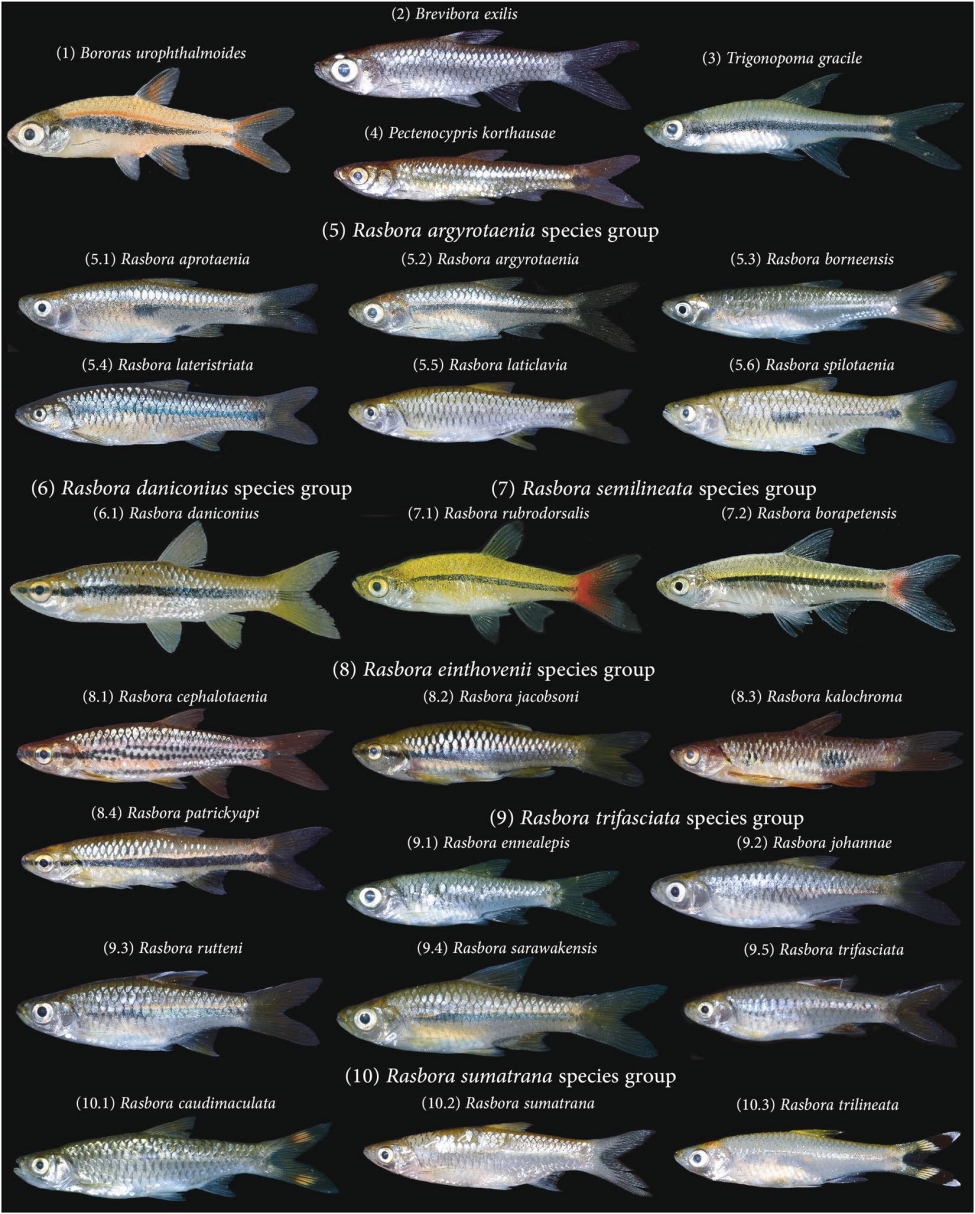
Selected species of Rasborinae that illustrate the diversity of the subfamily in Sundaland. All pictures, except 1, 6.1, 7.1 and 7.2, originate from the Barcode of Life Datasystem (dx.doi.org/110.5883/DS-BIFRA, Creative Commons Attribution - Non Commercial - Share Alike), pictures 1, 6.1 and 7.2 originate from FFish.asia (https://ffish.asia, Creative Commons Attribution - Non Commercial - Share Alike).

Species richness within vertebrate groups is high in Sundaland^1^ and freshwater fishes are no exceptions to that. With more than 900 species reported to date, and with nearly 45 percent of endemism, Sundaland’s ichthyofauna is the largest in SEA and accounts for nearly 75 percent of the entire ichthyodiversity of the Indonesian archipelago^15^. The inventory of Sundaland’s freshwater fishes started more than two centuries ago and despite the acceleration of species discovery over the last three decades, it is still a work in progress^15^. The complexity of this inventory was partly exacerbated by the abundance of minute species *i.e*. less than 5 cm length^15^, but also by the inconsistent use of species names through time for old descriptions due to the loss of type specimens or uncertainties in the location of type localities^16,17^. The family Cyprinidae *sensu lato* is a particularly good example for the complexity of Sundaland freshwater fishes taxonomy and systematics. The systematics of this large family of Cypriniformes, with over 3,000 species, has been controversial for more than a century^18^. Based on recent molecular phylogenetic studies^19–21^, Tan and Armbruster^22^ proposed a new classification dividing the Cyprinidae *sensu lato* into 12 families. The subfamily Rasborinae (Cypriniformes, Cyprinoidei, Danionidae) comprises roughly 140 species in 11 genera: *Amblypharyngodon*, *Boraras*, *Brevibora*, *Horadandia*, *Kottelatia*, *Pectenocypris*, *Rasbora*, *Rasboroides*, *Rasbosoma*, *Trigonopoma*, and *Trigonostigma*^22^. In Sundaland the subfamily Rasborinae is represented by 79 species in 7 genera. The genera *Amblypharyngodon*, *Horadandia*, *Rasboroides*, and *Rasbosoma* do not occur in Sundaland. By far the most species rich rasborine genus is *Rasbora* with over 100 species in total and 65 species in Sundaland. Long considered a catch-all group, several attempts have been made to provide a classification of the genus *Rasbora* that reflects phylogeny. In a comprehensive revision, Brittan^23^ recognized 3 subgenera (*Rasbora*, *Rasboroides*, and *Megarasbora*) and divided *Rasbora* into 8 species complexes, now regarded as species groups^24^ (Fig. 1). Subsequent authors have erected several new genera or suggested new species composition for the various *Rasbora* species groups^19,24–26^. Clearly, to better understand the evolutionary history of this unique group, the taxonomy and systematic of the Rasborinae needs to be better understood.

The use of standardized DNA-based approaches to the inventory of Sundaland’s ichthyofauna resulted in the detection of considerable knowledge gaps^16,17,27^. In addition, substantial levels of cryptic diversity (*i.e*. morphologically unrecognized diversity) were repeatedly reported for a wide range of Sundaland freshwater fish taxa^10,27–33^ including the Rasborinae^16^. The taxonomy of most Rasborinae species, particularly so for the genus *Rasbora*, remains challenging due to the diversity of the group and the morphological similarity of many closely related species. As a consequence, the actual distribution ranges of many species of Rasborinae are not well known.

This study aims to re-examine Rasborinae diversity in Sundaland. We generated a DNA barcode reference library to (1) explore biological species boundaries with DNA-based species delimitation methods, (2) validate species identity, taxonomy and precise range distribution by producing DNA barcodes from type localities or neighboring watersheds, (3) validate or revise of the previously published DNA barcodes records for the subfamily Rasborinae available on GenBank.

## Results

**Figure 2:**
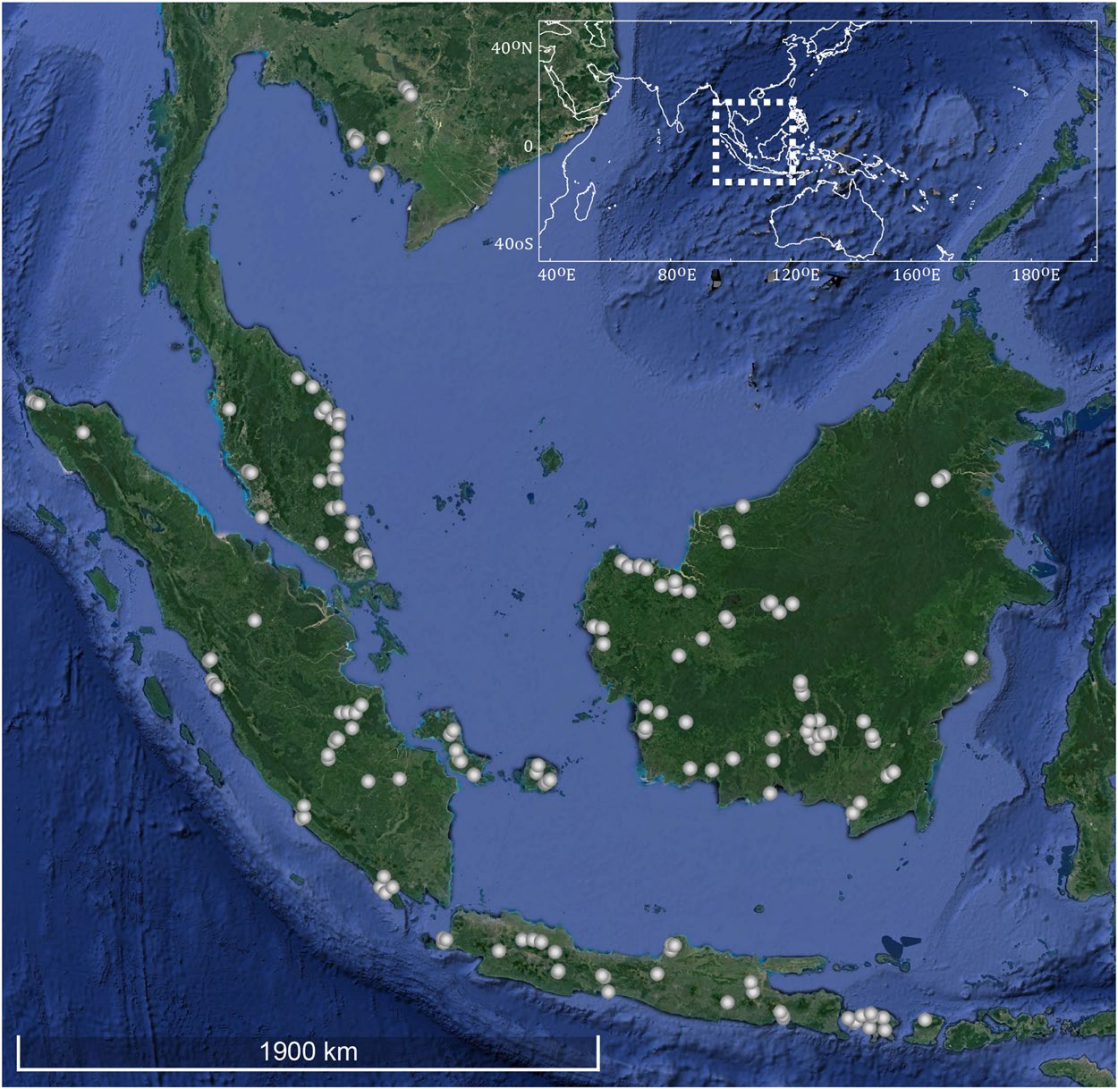
Collection sites for the newly generated 991 samples analyzed here. Each dot may represent several collection sites. Map data: Google, DigitalGlobe. Modified using Adobe Illustrator CS5 v 15.0.2. http://www.adobe.com/products/illustrator.html.

Sequencing of the DNA barcode marker Cytochrome Oxidase 1 (COI) yielded a total of 991 new sequences (Table S2) from 189 sampling sites distributed across Sundaland (Fig. 2). Together with 106 DNA barcodes mined from GenBank and BOLD (Table S3), we assembled a DNA barcode reference library of 1,097 sequences from 65 taxa of Rasborinae and 1 taxon of Sundadanionidae (*Sundadanio retiarius*). The number of specimens analyzed per species ranged from 1 to 143, with an average of 14.6 sequences per species and only six species represented by a single sequence. The sequences ranged from 459 bp to 651 bp long, with 99 percent of the sequences being above 500 bp length, and no stop codons were detected, suggesting that all the sequences correspond to functional mitochondrial COI sequences. DNA barcodes for 61 of the 79 nominal species of Rasborinae reported from Sundaland were recovered (approximately 78%) corresponding to the 7 Rasborinae genera currently recognized (Table S1). The present study achieved a complete coverage at the species level for the genera *Boraras* (2 species), *Brevibora* (3 species), *Kottelatia* (1 species), *Trigonopoma* (2 species) and *Trigonostigma* (3 species). In turn, two out of the three *Pectenocypris* species (66%) and 48 out of the 65 *Rasbora* species (74%) currently recognized in Sundaland were collected (Table S1). Geographically, our dataset includes 86% of the Rasborinae of Borneo (38 out of 44), all the *Rasbora* species of Java (4 species) and 68% of the Rasborinae species of Sumatra (26 out of 38) were collected (Table S1). Finally, four undescribed taxa are highlighted, two taxa of *Rasbora* in Java, one taxon of *Trigonostigma* in Borneo (Table S2) and an additional *Rasbora* taxon, previously assigned to *R. paucisqualis* in the literature (Table S3).

**Figure 3:**
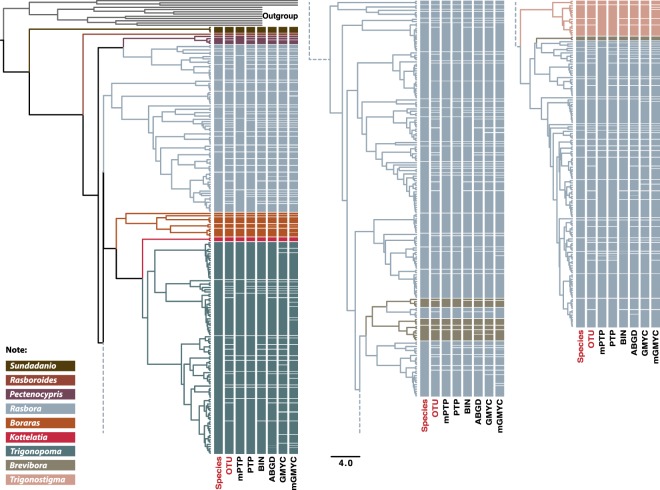
Bayesian maximum credibility tree of the Rasborinae DNA barcodes (identical haplotypes removed) and species delimitation according to GMYC, mGMYC, PTP, mPTP, ABGD, BIN and the 50% consensus delimitation.

Species delimitation analyses provided varying numbers of Operational Taxonomic Units (OTUs) among methods (Fig. 3): 129 for PTP, 95 for mPTP, 178 for GMYC, 191 for mGMYC, 175 for ABGD and 146 for RESL (Table S3). Our consensus delimitation scheme yielded 166 OTUs, including 165 OTUs for the 65 Rasborinae taxa, 2.5-fold more than by using morphological characters. The number of OTUs observed within species ranged from two for 22 species to 11 for *Trigonostigma pauciperforata* (Table 1). Based on the results of the species delimitation analyses, a re-examination of the original species identity associated with 105 DNA barcodes mined from BOLD and GenBank revealed 13 cases of conflicts that likely originated from mis-identifications (Table S4). These concerned the genera *Boraras* (four records, two species), *Brevibora* (two records, two species) and *Rasbora* (seven records, six species). Along the same line, 12 uncertain identifications were revised for the genera *Rasbora* (10 records, five taxa) and *Trigonostigma* (two records, one taxa).

**Table 1:** List of the morphological species displaying more than one OTU including the maximum intraspecific and minimum nearest neighbor K2P distances for species and OTUs.

Species/OTUs	Max. Intraspecfic Dist. (%)	Nearest Neighbor Dist. (%)
***Brevibora cheya***	4.29	5.99
OTU 105 (BOLD:AAY0408)	0.00	3.18
OTU 106 (BOLD:ADN0681)	1.30	3.18
***Brevibora dorsiocellata***	2.10	7.71
OTU 102 (BOLD:ADY4509)	0.00	1.57
OTU 103 (BOLD:ADN0680)	0.52	1.57
***Rasbora aprotaenia***	1.83	1.04
OTU 140 (**BOLD:ADY6054**)	1.30	1.04
OUT 139 (BOLD:ADZ0447)	NA	1.30
***Rasbora argyrotaenia***	5.67	5.97
OTU 87 (BOLD:ADY7291)	0.26	5.10
OTU 88 (BOLD:ACQ2593)	0.52	5.10
***Rasbora arundinata***	2.63	2.10
OTU 130 (BOLD:ADF6073)	0.00	2.10
OTU 131 (BOLD:ADN1040)	0.00	2.63
***Rasbora bankanensis***	7.12	6.51
OTU 40 (BOLD:ACF1059)	GenBank	GenBank
OTU 39 (BOLD:AAR2899)	0.00	1.30
OTU 38 (BOLD:ADY4700)	NA	1.30
OTU 41 (BOLD:ADY2504)	0.00	2.91
OTU 42 (BOLD:ADY1545)	0.00	3.72
OTU 43 (BOLD:ADY1544)	0.00	2.91
OTU 44 (BOLD:ACC0430)	1.04	1.57
OTU 144 (BOLD:ADY5341)	1.04	1.57
***Rasbora beauforti***	2.37	9.79
OTU 33 (**BOLD:ADY4385**)	NA	2.10
OTU 34 (**BOLD:ADY4385**)	0.26	2.10
***Rasbora borapetensis***	7.73	5.97
OTU 86 (BOLD:ADY1548)	NA	7.43
OTU 91 (BOLD:AAU5232)	0.52	5.97
***Rasbora caudimaculata***	1.83	7.71
OTU 100 (BOLD:ADO5236)	0.00	1.83
OTU 101 (BOLD:AAR2916)	NA	1.83
***Rasbora cephalotaenia***	6.84	7.68
OTU 4 (BOLD:ADY2668)	2.36	5.41
OTU 5 (BOLD:AAI0355)	GenBank	GenBank
OTU 6 (BOLD:ADN8441)	0.26	3.17
OTU 7 (BOLD:AAI0356)	0.78	3.17
***Rasbora daniconius***	0.26	11.18
OTU 2 (BOLD:ABX6594)	GenBank	GenBank
OTU 3 (BOLD:ACA0514)	0.26	11.18
***Rasbora dusonensis***	1.57	10.73
OTU 10 (BOLD:AAU2983)	0.26	1.30
OTU 9 (BOLD:ADN2767)	0.00	1.30
***Rasbora einthovenii***	11.10	8.31
OTU 73 (BOLD:ADY2667)	NA	7.45
OTU 74 (BOLD:ADY1546)	0.00	7.45
OTU 75 (BOLD:ADY1017)	0.00	7.75
OTU 77 (BOLD:ADW2748)	GenBank	GenBank
OTU 78 (BOLD:ADN0813)	0.00	5.43
OTU 79 (BOLD:AAU5112)	0.00	3.18
OTU 80 (BOLD:ADO6360)	NA	2.10
OTU 81 (BOLD:ADY1549)	1.57	2.10
OTU 83 (BOLD:ADY0551)	0.52	1.30
OTU 82 (BOLD:ADY0550)	0.78	1.30
***Rasbora elegans***	1.57	1.04
OTU 138 (**BOLD:ADY6054**)	0.00	1.04
OTU 136 (BOLD:ADY7956)	NA	1.30
OTU 137 (BOLD:ADZ0446)	0.00	1.30
***Rasbora ennealepis***	9.14	6.51
OTU 35 (BOLD:ADN3883)	0.00	5.94
OTU 36 (BOLD:ADN3887)	0.78	3.97
OTU 37 (BOLD:ADY4386)	0.26	3.97
***Rasbora jacobsoni***	0.00	8.84
OTU 66 (BOLD:ADW4597)	GenBank	GenBank
OTU 67 (BOLD:ADN9402)	0.00	8.84
***Rasbora kalbarensis***	0.52	12.42
OTU 20 (BOLD:AAY0409)	GenBank	GenBank
OTU 21 (BOLD:ADN1457)	0.52	12.42
***Rasbora kalochroma***	2.64	5.39
OTU 71 (**BOLD:AAR2898**)	NA	1.30
OTU 72 (**BOLD:AAR2898**)	1.83	1.30
***Rasbora kottelati***	2.37	5.39
OTU 68 (BOLD:ADX8298)	GenBank	GenBank
OTU 69 (BOLD:ADN0290)	0.00	2.10
OTU 70 (BOLD:ADX9355)	0.26	2.10
***Rasbora lateristriata***	1.83	2.90
OTU 141 (BOLD:ACQ7159)	1.30	1.57
OTU 142 (BOLD:ACQ7160)	0.00	1.57
***Rasbora laticlavia***	4.55	4.00
OTU 119 (BOLD:ADN8626)	NA	3.45
OTU 120 (BOLD:ADO3612)	0.78	1.04
OTU 121 (BOLD:ADY6696)	0.26	1.04
***Rasbora patrickyapi***	1.83	8.31
OTU 146 (**BOLD:ADN2766**)	0.00	1.83
OTU 76 (**BOLD:ADN2766**)	0.00	1.57
OTU 147 (**BOLD:ADN2766**)	NA	1.57
***Rasbora paucisqualis***	5.97	7.63
OTU 26 (BOLD:ADY2665)	0.00	4.28
OTU 27 (BOLD:ADX9120)	0.00	2.63
OTU 28 (BOLD:ADY4316)	NA	1.57
OTU 29 (BOLD:ADY4315)	NA	1.57
OTU 30 (BOLD:ADY4317)	0.26	1.83
***Rasbora paviana***	2.37	2.36
OTU 126 (**BOLD:AAD6182**)	0.52	1.83
OTU 127 (**BOLD:AAD6182**)	GenBank	GenBank
OTU 129 (BOLD:ADY6053)	1.30	1.83
***Rasbora rutteni***	6.22	7.14
OTU 17 (BOLD:ADN4430)	1.04	5.93
OTU 18 (BOLD:ADY4516)	0.00	2.37
OTU 19 (BOLD:ADN7331)	0.00	2.37
***Rasbora sp.1***	2.63	3.45
OTU 124 (BOLD:ACQ2698)	0.00	2.63
OTU 125 (BOLD:ACQ2594)	0.52	2.63
***Rasbora subtilis***	3.99	7.06
OTU 111 (BOLD:ADN7332)	NA	3.99
OTU 112 (BOLD:ADN3888)	1.57	3.99
***Rasbora sumatrana***	3.18	5.97
OTU 89 (**BOLD:AAY0407**)	1.04	2.37
OTU 90 (**BOLD:AAY0407**)	0.78	2.37
***Rasbora tornieri***	1.57	9.51
OTU 84 (**BOLD:ADL5624**)	NA	1.57
OTU 85 (**BOLD:ADL5624**)	0.00	1.57
***Rasbora trilineata***	10.97	7.69
OTU 96 (BOLD:AAE7383)	1.83	2.36
OTU 97 (BOLD:ADN9095)	0.00	5.96
OTU 98 (BOLD:ADN7260)	NA	3.74
OTU 99 (BOLD:ADN9096)	0.00	3.74
OTU 93 (**BOLD:AAE7384**)	GenBank	GenBank
OTU 94 (**BOLD:AAE7384**)	0.00	4.27
OTU 95 (BOLD:ADY1696)	0.26	2.36
***Rasbora tuberculata***	9.87	9.73
OTU 24 (BOLD:ADN3886)	0.00	9.55
OTU 25 (BOLD:ADN3884)	0.26	9.55
***Rasbora vaillantii***	1.83	4.00
OTU 117 (BOLD:ADY8198)	0.00	1.83
OTU 118 (BOLD:ADY8199)	0.00	1.83
***Rasbora vulcanus***	0.00	7.69
OTU 115 (BOLD:AAI0352)	GenBank	GenBank
OTU 116 (BOLD:ADN3885)	0.00	7.69
***Trigonopoma gracile***	13.64	9.22
OTU 46 (BOLD:ADN4644)	NA	6.26
OTU 47 (BOLD:ADO0069)	0.00	1.57
OTU 48 (BOLD:ADY2669)	NA	2.36
OTU 49 (BOLD:ADY4282)	NA	1.57
OTU 50 (BOLD:ADY6176)	0.00	1.57
OTU 145 (**BOLD:ACC0899**)	0.52	2.10
OTU 51 (**BOLD:ACC0899**)	2.10	2.10
***Trigonopoma pauciperforatum***	9.25	9.22
OTU 55 (BOLD:ADY1547)	1.83	1.83
OTU 56 (BOLD:ADY1425)	0.00	1.83
OTU 57 (BOLD:ADY5548)	0.52	2.10
OTU 58 (BOLD:AAV7972)	NA	4.81
OTU 59 (BOLD:ACC0580)	1.30	4.54
OTU 60 (**BOLD:ADY2666**)	0.00	1.30
OTU 61 (**BOLD:ADY2666**)	1.57	1.30
OTU 62 (BOLD:ADN4643)	NA	3.45
OTU 63 (BOLD:ACC0669)	0.00	3.99
OTU 64 (BOLD:ADV1540)	2.37	1.83
OTU 65 (BOLD:AAY0427)	1.83	1.83
***Trigonostigma heteromorpha***	2.37	2.64
OTU 108 (BOLD:AAJ8936)	0.26	1.83
OTU 110 (BOLD:ABZ6147)	0.26	1.83

**Figure 4:**
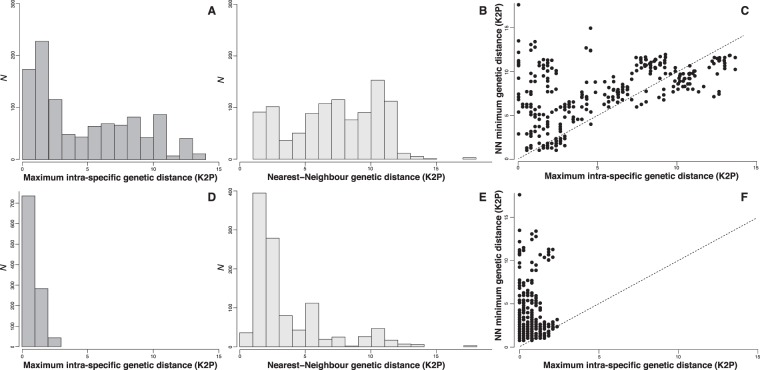
Summary of the distribution of the K2P distances. (**A**,**D)** Maximum intraspecific K2P distances; (**B**,**E)** Minimum nearest-neighbor K2P distances; (**C**,**F)** Individual plotting of maximum intraspecific K2P distances and minimum nearest neighbor K2P distances. (**A**–**C)** Distributions of K2P distances for species delimitated using morphological characters. (**D**–**F)** Distributions of K2P distances for OTUs delimitated by the 50% consensus among species delimitation methods.

The examination of the maximum K2P genetic distances for species with multiple OTUs and within OTUs revealed large differences with maximum K2P distances ranging between 0.26 and 13.64 within species and between 0.00 and 2.37 within OTUs (Table 1). This trend was largely confirmed by the distribution of the genetic distances at both species and OTUs levels (Fig. 4). At the species level, the distribution of the maximum intraspecific K2P genetic distance broadly overlap with the distribution of the K2P distances to the nearest neighbor (Fig. 4A,B, Table S5) and no barcoding gap is observed. On average, the nearest neighbor K2P genetic distances are only 3.5-fold higher than the maximum intraspecific K2P distances. Plotting genetic distance for each species provides little improvement as a substantial number of species display maximum intraspecific K2P genetic distances higher than the minimum distance to the nearest neighbor (Fig. 4C). At the OTU level, the overlap is drastically reduced peaking between 0 and 0.99 for maximum intraspecific K2P distances and ranging between 1.0 and 1.99 for the K2P distance to the nearest neighbor (Fig. 4D,E). The distribution width of the maximum intraspecific K2P distance is much more restricted for OTUs than species and fewer cases of maximum intraspecific distance higher than the minimum distance to the nearest neighbor are observed (Fig. 4F). At the OTU level, the nearest neighbor K2P genetic distances were 7.2-fold higher on average than the maximum intraspecific K2P genetic distances.

**Figure 5:**
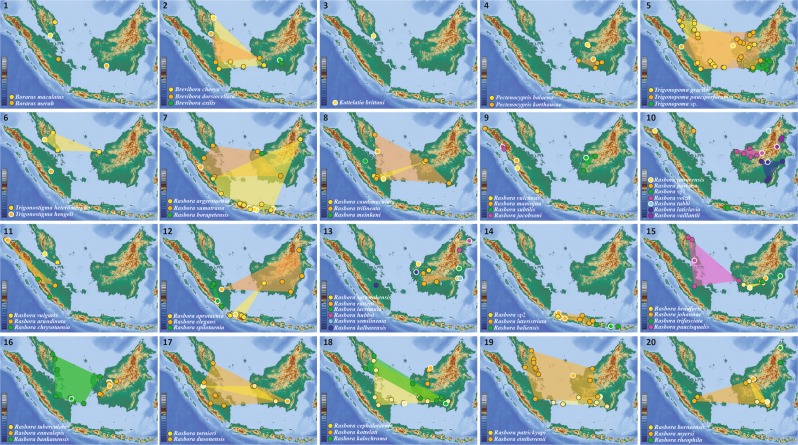
Maps depicting species distribution ranges as established based on the present sampling sites (black margin) and type localities (white margin) following the checklist generated for this study (Table S1). **1** Sampling sites and type localities of *Boraras maculatus* and *B. merah*. **2** Sampling sites, type localities and distribution ranges of *Brevibora cheeya*, *B. dorsiocellata* and *B. exilis*. **3** Type locality of *Kottelatia brittani*, sampling sites unknown, sequences originating from GenBank. **4** Sampling sites, type localities and distribution ranges of *Pectenocypris korthausea* and *P. balaena*. **5** Sampling sites, type localities and distribution ranges of *Trigonopoma gracile*, *T. pauciperforatum* and *T*. sp^16^. **6** Sampling sites, type localities and distribution ranges of *Trigonostigma heteromorpha*, *T. hengeli* (sampling sites outside the map); *T. espei* not displayed, type locality outside the map and sampling sites unknown, sequences originating from GenBank. **7** Sampling sites, type localities and distribution ranges of *Rasbora argyrotaenia*, *R. sumatrana* and *R. borapetensis*, multiple type localities for *Rasbora argyrotaenia* as detailed in^16^, Type locality of *R. borapetensis* outside the map. **8** Sampling sites, type localities and distribution ranges of *Rasbora caudimaculata*, *R. trilineata* and *R. meinkeni*, sampling sites of *R. meinkeni* unknown, sequence originating from GenBank. **9** Sampling sites, type localities and distribution ranges of *Rasbora vulcanus*, *R. maninjau*, *R. subtilis* and *R. jacobsoni*. **10** Sampling sites, type localities and distribution ranges of *Rasbora tawarensis*, *R. paviana*, R. sp. 1^16^, *R. volzii*, *R. tubbi*, *R. laticlavia* and *R. vaillanti*, sampling sites corresponding to the type locality for *Rasbora tawarensis*. **11** Sampling sites, type localities and distribution ranges of *Rasbora vulgaris*, *R. arundinata* and *R. chrysotaenia*, type locality of *Rasbora chrysotaenia* located in Sumatra with no further details (Table S1). **12** Sampling sites, type localities and distribution ranges of *Rasbora aprotaenia*, *R. elegans* and *R. spilotaenia*. **13** Sampling sites, type localities and distribution ranges of *Rasbora sarawakensis*, *R. rutteni*, *R. lacrimula*, *R. hubbsi*, *R. semilineata* and *R. kalbarensis*. **14** Sampling sites, type localities and distribution ranges of *Rasbora* sp2^16^, *R. lateristriata* and *R. baliensis*. **15** Sampling sites, type localities and distribution ranges of *Rasbora beauforti*, *R. johannae*, *R. trifasciata* and *R. paucisqualis*. **16** Sampling sites, type localities and distribution ranges of *Rasbora tuberculata*, *R. ennealepis* and *R. bankanensis*. **17** Sampling sites, type localities and distribution ranges of *Rasbora tornieri* and *R. dusonensis*. **18** Sampling sites, type localities and distribution ranges of *Rasbora cephalotaenia*, *R. kottelati* and *R. kalochroma*. **19** Sampling sites, type localities and distribution ranges of *Rasbora patrickyapi* and *R. einthovenii*. **20** Sampling sites, type localities and distribution ranges of *Rasbora borneensis*, *R. myersi*, *R. rheophila*. Sampling sites and type locality of *R. daniconius* not displayed, outside the map. Each locality may represent several sampling sites. Map data: https://maps-for-free.com/. Modified using Adobe Illustrator CS5 v 15.0.2. http://www.adobe.com/products/illustrator.html.

Range distributions inferred from the new records generated for this study indicate that most type localities are embedded in the observed species range (Fig. 5). The degree of overlap between species range, however, largely varies among genera with little or no overlap observed for *Boraras*, *Pectenocypris*, *Trigonostigma* and most *Rasbora* species while a substantial amount of overlap is observed for *Brevibora* and *Trigonopoma* species (Fig. 5).

## Discussion

This study represents the most comprehensive molecular survey conducted for the subfamily Rasborinae^19,34^. Our DNA barcode reference library consists of 65 Rasborinae species distributed across 7 genera and covering 78% of the Rasborinae diversity reported from Sundaland. DNA barcoding delivers reliable species-level identifications when taxa possess unique COI sequence clusters characterized by multiple private mutations. This condition was met for all the Rasborinae species examined here and no cases of retention of ancestral polymorphism were detected^35^. However, this clearly contrasts with multiples discrepancies observed within the set of previously published COI sequences obtained on GenBank and BOLD. About 25 percent of these records were either misidentified or associated with uncertain identifications. Such mis-identifications were expected considering the morphological uniformity within some Rasborinae genera, particularly in the genus *Rasbora* where multiple cases of taxonomic conflicts have been highlighted already^16,36–39^. Unexpectedly, most of the conflicts we detected were within the larger species of *Rasbora*, particularly those of the *Rasbora argyrotaenia* group and the *R. sumatra* group, and not within closely related smaller species such as members of the *R. trifasciata* group (Fig. 1). In facts, conflicts in species level population assignments have been previously reported for the *R. argyrotaenia* group in Java and Bali where *R. lateristriata* and *R. baliensis* have been confounded for decades as recently revealed by re-examination of species boundaries and distribution through DNA barcodes^16^. Other morphologically similar species of the *Rasbora argyrotaenia* group have been previously confused with *R. lateristriata*, such as *R. elegans*, *R. spilotaenia* and *R. chrysotaenia*. These species are difficult to separate due to overlapping meristic counts and coloration patterns^40^. Our study, however, highlights that these species have disjunct range distributions (Fig. 5) and cluster into well-differentiated mitochondrial lineages (Fig. S1, Table S3). Several of the detected misidentifications also involve species from different *Rasbora* species groups^24^ such as *Rasbora dusonensis*, from the *R. argyrotaenia* group, that has been previously mistaken for *R. sumatrana* from the *sumatrana* group and *R. myersi*, from the *R. sumatrana* group, that has been confounded with *R. dusonensis* from the *argyrotaenia* group. Despite being distantly related (Fig. S1), these species show overlapping meristic counts and similar coloration patterns with no dark spots on the body^40^. This result further calls for a broader assessment of the monophyly of the different *Rasbora* groups, previously identified by Liao^24^, as they are poorly supported by our study (Fig. S1).

The observed average ratio of 3.5 between intraspecific and interspecific distances is very low compared to earlier values found for the Javanese ichtyofauna, where minimum nearest neighbor genetic distances are on average 28-fold higher than the maximum intraspecific genetic distances^27^. This value is also very low in comparison to previous large-scale fish DNA barcode surveys^41–46^. This deviation can be attributed to a substantial amount of cryptic diversity revealed by our species delimitation analyses. For 61 species, delimitated on the basis of morphological characters, and validated by a match between species range distributions and type localities, we recovered a total of 166 OTUs. When accounting for this cryptic diversity the ratio between the minimum nearest neighbor and maximum intraspecific distances rose to 7.5. Earlier large scale surveys in Sundaland already pointed to substantial levels of cryptic diversity^28–31,33^ and it has also been demonstrated that small-size species are more sensitive to fragmentation, experience faster genetic drift and as such accumulate cryptic diversity at a faster rate than large-size species^45,47^. Along the same line, small-size species are more frequently confounded and lumped together, a bias that tend to inflate the proportion of hidden diversity^48^.

We found very high numbers of OTUs with deep genetic divergences (up to 13.64% in *Trigonopoma gracile*) in a number of species (ranging from 7 to 11) such as in *Rasbora bankanensis*, *Rasbora einthovenii*, *Rasbora trilineata*, *Trigonopoma gracile* and *Trigonopoma pauciperforatum*. These five species also display some of the widest range distributions in Sundaland with OTUs occurring in Borneo, Sumatra, Peninsular Malaysia and several small islands across the Java sea (*R. bankanensis*, Fig. 5(16); *R. einthovenii*, Fig. 5(19); *R. trilineata*, Fig. 5(8); *T. gracile*, Fig. 5(5); *T. pauciperforatum*, Fig. 5(4)). However, the scarcity of OTU range overlap for those species suggests ongoing population fragmentation across the species range distribution (Tables S2 and S3). This pattern is likely connected to the complex geological history of Sundaland which over the last 10 Million years was influenced by the subduction activity of the Asian and Australian plates and the resulting intense volcanic activity which produced multiple volcanic arches^5^. Furthermore, climatic fluctuations during the Pleistocene induced major sea levels changes leading to merging of Sundaland landmasses during glacial maxima and multiple fragmentations during glacial sea level low-stands^7,8^. In such dynamic landscapes, complex patterns of distribution and high lineage diversity are to be expected^10^. The influence of eustatic fluctuations in Sundaland is exemplified by *Rasbora bankanensis*, *Rasbora einthovenii*, *Rasbora trilineata*, *Trigonopoma gracile* and *Trigonopoma pauciperforatum* all of which display wide range distributions among watersheds neighboring the Java sea. Those have been repeatedly connected during glacial maxima (Fig. 5(5), 5(8), 5(16) and 5(19)). This pattern strongly contrasts with the lower genetic diversity and restricted range distribution of the species occurring in the Eastern part of Borneo such as *Rasbora vaillantii* (Fig. 5(10)), *R. laticlavia* (Fig. 5(10)), *R. trifasciata* (Fig. 5(15)) and *R. reophila* (Fig. 5(20)) or species occurring in the Western part of Sumatra such as *Rasbora vulcanus* (Fig. 5(9)), *R. maninjau* (Fig. 5(9)), *R. jacobsoni* (Fig. 5(9)), *R. tawarensis* (Fig. 5(10)); *R. chrysotaenia* (Fig. 5(11)) and *R. arundinata* (Fig. 5(11)) and species in Java and Bali such as *Rasbora* sp1 (Fig. 5(10)), R. sp2 (Fig. 5(14)), *R. lateristriata* (Fig. 5(14)) and *R. baliensis* (Fig. 5(14)). These parts of Borneo, Sumatra and partially Java were disconnected from the central region of Sundaland around the Java sea during the Pleistocene. This trend highlights the sensitive status of the endemic Rasborinae species in the peripheral areas of Sundaland due to their highly restricted distribution ranges. The present study also argues against translocation programs for the most widespread species, considering the high proportion of cryptic diversity, if species and OTUs identity are not determined through DNA barcodes^16,31^.

## Conclusions

The subfamily Rasborinae is the most diverse freshwater fish group of Sundaland and therefore represents an excellent model to explore the evolutionary response of local freshwater biotas to a dynamic geological history and repeated eustatic fluctuations. Affected by taxonomic confusions for decades, the genus *Rasbora* has been left aside of recent large-scale molecular studies aimed at exploring the diversification of aquatic biotas in Sundaland. Our comprehensive DNA barcode reference library for the subfamily enables further evolutionary studies on the diversification of the group, in particular within the genus *Rasbora*, which allowed us to trace evolutionary dynamics at the local scale in Sundaland^16^. The contrasting patterns of molecular diversity and species range distributions between Rasborinae species inhabiting the watersheds neighboring the Java sea and the species located on the Eastern part of Borneo call for a larger assessment of their dynamics of species proliferation based on broader genomic analyses. Clearly, future studies will also have to address the systematics of the Rasborinae as no evidence supporting the monophyly of *Rasbora* nor the different *Rasbora* species groups are detected here.

## Material and Methods

### Sampling and collection management

Material used in the present study is the result of a collective effort to assemble a global Rasborinae DNA barcode reference library through various field sampling efforts conducted by several of the coauthors in Sundaland over the past decade. Specimens were captured using gears such as electrofishing, seine nets, cast nets and gill nets across sites that encompass the diversity of freshwater lentic and lotic habitats in Sundaland (Fig. 2). Specimens were identified following original descriptions where available, as well as monographs^40,49^. Species names were further validated using several online catalogs^50,51^. Specimens were photographed, individually labeled and voucher specimens were preserved in a 5% formalin solution. Prior to fixation a fin clip or a muscle biopsy was taken and fixed separately in a 96% ethanol solution for further genetic analyses. Both tissues and voucher specimens were deposited in the national collections at the Museum Zoologicum Bogoriense (MZB), Research Center for Biology (RCB), Indonesian Institute of Sciences (LIPI).

### Assembling a checklist of the Sundaland Rasborinae

A checklist of the Rasborinae species occurring in Sundaland was assembled from available online catalogs including Fishbase^51^ and Eschmeyer’s Catalog of Fishes^50^ as detailed in Hubert *et al*.^15^. This checklist was used to estimate the taxonomic coverage of the present DNA barcoding campaign and to identify type localities for each species. The following information was included: (1) authors of the original description, (2) type locality, (3) latitude and longitude of the type locality, (4) holotype and paratypes catalog numbers, (5) distribution in Sundaland. This information is available as online Supplementary Material (Table S1).

### Sequencing and international repositories

Genomic DNA was extracted using a Qiagen DNeasy 96 tissue extraction kit following manufacturer’s specifications. A 651-bp segment from the 5′ region of the cytochrome oxidase I gene (COI) was amplified using primers cocktails C_FishF1t1/C_FishR1t1 including M13 tails^52^. PCR amplifications were done on a Veriti 96-well Fast (ABI-AppliedBiosystems) thermocycler with a final volume of 10.0 μl containing 5.0 μl Buffer 2× 3.3 μl ultrapure water, 1.0 μl each primer (10 μM), 0.2 μl enzyme Phire Hot Start II DNA polymerase (5U) and 0.5 μl of DNA template (~50 ng). Amplifications were conducted as followed: initial denaturation at 98 °C for 5 min followed by 30 cycles denaturation at 98 °C for 5 s, annealing at 56 °C for 20 s and extension at 72 °C for 30 s, followed by a final extension step at 72 °C for 5 min. The PCR products were purified with ExoSap-IT (USB Corporation, Cleveland, OH, USA) and sequenced in both directions. Sequencing reactions were performed using the “BigDye Terminator v3.1 Cycle Sequencing Ready Reaction” and sequencing was performed on the automatic sequencer ABI 3130 DNA Analyzer (Applied Biosystems). DNA barcodes obtained at the Naturhistorisches Museum Bern were generated as previously described in Conte-Grand *et al*.^33^.

The sequences and associated information were deposited on BOLD^53^ and are available in the data set DS-BIFRA (Table S2, dx.doi.org/10.5883/DS-BIFRA). DNA sequences were submitted to GenBank (accession numbers are accessible directly at the individual records in BOLD). An additional set of 106 Rasborinae COI sequences were downloaded from GenBank (Table S3).

### Genetic distances and species delimitation

Kimura 2-parameter (K2P)^54^ pairwise genetic distances were calculated using the R package Ape 4.1^55^. Maximum intraspecific and nearest neighbor genetic distances were calculated from the matrice of pairwise K2P genetic distances using the R package Spider 1.5^56^. We checked for the presence of a barcoding gap, *i.e*. the lack of overlap between the distributions of the maximum intraspecific and the nearest neighbor genetic distances^57^, by plotting both distances and examining their relationships on an individual basis instead of comparing both distributions independently^58^. A neighbor-joining (NJ) tree was built based on K2P distances using PAUP 4.0a^59^ in order to visually inspect genetic distances and DNA barcode clusters (Fig. S1). This NJ tree was rooted using *Sundadanio retiarius*.

Several alternative methods have been proposed for delimitating molecular lineages^60–63^. Each of these methods have pitfalls, particularly when it comes to singletons (*i.e*. delimitated lineages represented by a single sequence) and a combination of different approaches is increasingly used to overcome potential pitfalls arising from uneven sampling^16,43,64–66^. We used four different sequence-based methods of species delimitation. For the sake of clarity, we refer to species identified based on morphological characters as species while species delimited using DNA sequences are referred to as Operational Taxonomic Unit (OTU)^67–69^. OTUs were delimitated using the following algorithms: (1) Refined Single Linkage (RESL) as implemented in BOLD and used to generate Barcode Index Numbers (BIN)^62^, (2) Automatic Barcode Gap Discovery (ABGD)^61^, (3) Poisson Tree Process (PTP) in its multiple rates version (mPTP) as implemented in the stand-alone software mptp_0.2.3^63,70^, (4) General Mixed Yule-Coalescent (GMYC) in its multiple rate version (mGMYC) as implemented in the R package Splits 1.0–19^71^. RESL and ABGD used DNA alignments as input files while a ML tree was used for mPTP and a Bayesian Chronogram based on a strict-clock model using a 1.2% of genetic distance per million year^72^ for mGMYC. The mPTP algorithm uses a phylogenetic tree as an input file, thus, a maximum likelihood (ML) tree was first reconstructed using RAxML^73^ based on a GTR + Γ substitution model. Then, an ultrametric and fully resolved tree was reconstructed using the Bayesian approach implemented in BEAST 2.4.8^74^. Two Markov chains of 50 millions each were ran independently using the Yule pure birth model tree prior, a strict-clock model and a GTR + I + Γ substitution model. Trees were sampled every 10,000 states after an initial burnin period of 10 millions. Both runs were combined using LogCombiner 2.4.8 and the maximum credibility tree was constructed using TreeAnnotator 2.4.7^74^. Identical haplotypes were pruned for further species delimitation analyses.

## Supplementary information

Supplementary Information.

Supplementary Information 2.

Supplementary Information 3.

Supplementary Information 4.

Supplementary Information 5.

Supplementary Information 6.

Supplementary Information 7.
