# Supplementary material for: Disentangling the taxonomy of the subfamily Rasborinae (Cypriniformes, Danionidae) in Sundaland using DNA barcodes
Source: Sci Rep. 2020 Feb 18;10:2818. doi: 10.1038/s41598-020-59544-9 (PMC7028728; doi:10.1038/s41598-020-59544-9)
Supplement: Supplementary file 2 — Supplementary Information 2. [file 41598_2020_59544_MOESM2_ESM.pdf]

Disentangling the taxonomy of the subfamily Rasborinae (Cypriniformes, Danionidae) in Sundaland using DNA barcodes.

Arni Sholihah[1,2], Erwan Delrieu-Trottin[1,3], Tedjo Sukmono[4], Hadi Dahruddin[1,5], Renny Risdawati[6], Roza Elvyra[7], Arif Wibowo[8,9], Kustiati Kustiati[10], Frédéric Busson[2,11], Sopian Sauri[5], Ujang Nurhaman[5], Edmond Dounias[12], Muhamad Syamsul Arifin Zein [5], Yuli Fitriana[5], Ilham Vemendra Utama[5], Zainal Abidin Muchlisin[13], Jean-François Agnèse[1], Robert Hanner[14], Daisy Wowor[5], Dirk Steinke[14], Philippe Keith[11], Lukas Rüber[15,16], Nicolas Hubert[1].

[1]Instut Teknologi Bandung, School of Life Sciences and Technology, Bandung, Indonesia.

[2]UMR 5554 ISEM (IRD, UM, CNRS, EPHE), Université de Montpellier, Place Eugène Bataillon, 34095 Montpellier cedex 05, France.

[3]Museum für Naturkunde, Leibniz-Institut für Evolutions und Biodiversitätsforschung an der Humboldt-Universität zu Berlin, Invalidenstrasse 43, Berlin 10115, Germany

[4]Universitas Jambi, Department of Biology, Jalan Lintas Jambi - Muara Bulian Km15, 36122 Jambi, Sumatra, Indonesia.

[5]Division of Zoology, Research Center for Biology, Indonesian Institute of Sciences (LIPI), Jalan Raya Jakarta Bogor Km 46, Cibinong 16911, Indonesia.

[6]Department of Biology Education, STKIP PGRI Sumatera Barat, Jl Gunung Pangilun, Padang 25137, Indonesia

[7]Universitas Riau, Department of Biology, Simpang Baru, Tampan, Pekanbaru 28293, Indonesia

[8]Southeast Asian Fisheries Development Center, Inland Fisheries Resources Development and Management Department, 8 Ulu, Seberang Ulu I, Palembang 30267, Indonesia

[9] Research Institute for Inland Fisheries and Fisheries extensions, Agency for Marine and Fisheries Research, Ministry of Marine Affairs and Fisheries., Jl. H.A. Bastari No. 08, Jakabaring, Palembang 30267, Indonesia

[10]Universitas Tanjungpura, Department of Biology, Jalan Prof. Dr. H. Hadari Nawawi, Pontianak 78124, Indonesia

[11]UMR 7208 BOREA (MNHN-CNRS-UPMC-IRD-UCBN), Muséum National d'Histoire Naturelle, 43 rue Cuvier, 75231 Paris cedex 05, France.

[12]UMR 5175 CEFE (IRD, UM, CNRS, EPHE), 1919 route de Mende, 34293 Montpellier cedex 05, France.
[13]Syiah Kuala University, Faculty of Marine and Fisheries, Banda Aceh, 23111, Indonesia
[14]Department of Integrative Biology, Centre for Biodiversity Genomics, 50 Stone Rd E, Guelph, ON N1G2W1, Canada
[15]Naturhistorisches Museum Bern, Bernastrasse 15, Bern 3005, Switzerland
[16]Aquatic Ecology and Evolution, Institute of Ecology and Evolution, University of Bern, 3012 Bern, Switzerland

Corresponding author: Nicolas Hubert
Corresponding author email: nicolas.hubert@ird.fr

Supplementary information

Figure S1. Neighbor joining clustering tree for all 1097 Rasborinae DNA barcodes.

Table S1. Checklist of Rasborinae species occurring in Sundaland including authors, type localities, latitude and longitude of the type localities, holotypes collection numbers, paratypes collection numbers, maximum length, maximum length descriptor, occurrences in Bali, Banka, Batam/Bintang, Belintong, Buru, Java, Kalimantan, Madura, Natuna/Riau, Sumatra, Peninsular Malaysia and inclusion in the present study.

Table S2. Collateral information of the 991 newly generated DNA barcodes.

Table S3. Collateral information for the 105 previously published DNA barcodes originating from GenBank and BOLD including accession numbers, original identification, status based on the re-examination of the sample identity and updated identifications.

Table S4. Results of the species delimitation analyses including GMYC, mGMYC, PTP, mPTP, ABGD, BIN and 50% consensus among the six methods.

Table S5. Barcode gap analysis including maximum and average intraspecific K2P distances and minimum K2P distance to the nearest neighbor as computed in BOLD for the 991 newly generated DNA barcodes.
