# Supplementary material for: Disentangling the taxonomy of the subfamily Rasborinae (Cypriniformes, Danionidae) in Sundaland using DNA barcodes
Source: Sci Rep. 2020 Feb 18;10:2818. doi: 10.1038/s41598-020-59544-9 (PMC7028728; doi:10.1038/s41598-020-59544-9)

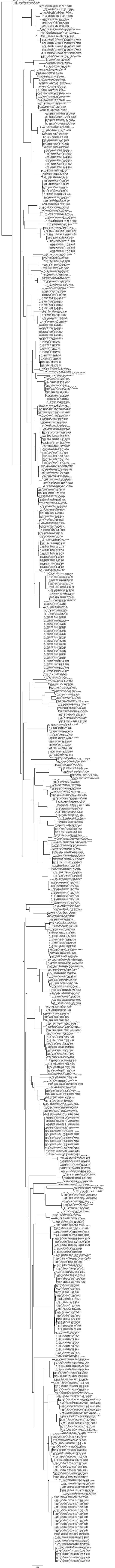

Supplement: Supplementary file 1 — Supplementary Information. [file 41598_2020_59544_MOESM1_ESM.pdf]
